# Disappearing male sterilization in India: do we care?

**DOI:** 10.1186/s40834-023-00228-w

**Published:** 2023-05-04

**Authors:** Pradeep S. Salve, Chander Shekhar

**Affiliations:** 1 Department of Population & Development, International Institute for Population Sciences, Mumbai, 400088 India; 2 Department of Fertility & Social Demography, International Institute for Population Sciences, Mumbai, 400088 India

**Keywords:** Family Planning, Health Policy, Sterilization, National Family Health Survey (NFHS), India

## Abstract

The disproportionate share of contraceptive use by women makes family planning services women-centric in India. The meagre and declining share of male-based contraceptive use, along with the overwhelming prevalence of female sterilization, has its underpinning in the lack of emphasis on fertility regulation with the use of temporary methods. The recent trends in the share of contraceptive choices not only contest the most touted cafeteria approach but also serves counter to the National Health Policy target of increasing vasectomy prevalence. Hence an inspection of trends in vasectomy may help in course correction needed to realise NHP targets.

**Supplementary Information:**

The online version contains supplementary material available at 10.1186/s40834-023-00228-w.

## Introduction

Women’s reproductive health and rights occupied the centre stage at the International Conference on Population and Development (ICPD)-1994 in Cairo. It assured that women have complete freedom to opt for different types of family planning methods to ensure safe pregnancy and motherhood and access to child health services. Globally, ICPD was a milestone and made substantial changes in shaping the perspective of policymakers and programme managers and their attitudes towards women’s sexual life and reproductive and maternal health. Several countries adopted ICPD resolutions and initiated couple-centric welfare programmes. Following the ICPD programme of action, India discontinued the target-orientated family planning programmes and services throughout the country, replacing them with a cafeteria^1^ approach to contraceptive provisioning. It further guaranteed that women would receive quality adolescent, reproductive, maternal, and child health services through different programmes in all public health facilities. However, despite many women-centric welfare programme initiatives, there is a skewed use pattern of family planning with an undue share of women-specific methods without any sign of convergence. The Sustainable Development Goal—5 (SGD-5) promises to achieve gender equality and end all forms of discrimination against all women and girls everywhere. However, a systematic female bias has been observed while considering the utilization of sterilization services in India. The section-4.8 of the National Health Policy (NHP)—2017 aims to increase the proportion of male sterilization from less than 5 percent to at least 30 percent and, if possible, much higher [1]. This unrealistic target of NHP for increasing male sterilization (vasectomy) seems far from realization without a systematic roadmap of intervention. As of now, India has few programmatic incentives that encourage people to adopt vasectomy, including conditional cash incentives and service increments [2].

In fact, India was the first country in the world to implement a family planning programme officially throughout the country in 1952. Initially, the clinical-based approach was introduced to deliver family planning services which suffered underutilization of services by people owing to the social stigma attached to it. The permanent family planning method (male sterilization/vasectomy) was common among males up to the 1980s in India [3]. For instance, 80.5 percent of total sterilization were vasectomies during 1966–70 in India. However, subsequently, it decreased from 65.1 percent in 1971–75 to 14.8 percent in 1981–85 [4, 5]. During 1986–90 only 13.4 percent of total sterilizations were vasectomies which further reduced to 3.4 percent and 1.9 percent of total sterilization in 1998–99 [5–8]. According to the NFHS-5, the share of vasectomy in current contraceptive use remains less than 1 percent (0.3%) in India (NFHS-5) [9]. A similar pattern holds true in in-service utilization statistics from Health Management Information System (HMIS), evidence of the stark disparity where about 55,324 male sterilizations were conducted in India during 2019–20 compared to 34,02,458 female sterilizations [10].

The coercive, politically motivated family planning camp-based approach during 1975–77 resulted in a steady decrease in vasectomies in India. For instance, total 76,37,495 vasectomy operations were performed during the emergency period (1975–77) when compared to the pre-emergency (6,11,960) in 1974–75 and post-emergency (1,87,609) in 1977–78 (Supplementary Table 1). After the failure of the camp approach, positive and negative incentives, and compulsory sterilization leads to shift towards the acceptance of permanent family planning which led to the popularisation of female sterilization methods. Five rounds of NFHS reported that about two-thirds of women were sterilized in India. Female sterilization has become the most socially accepted phenomenon in Indian society today. Henceforth, the increasing share of male sterilization, as highlighted by NHP-2017 is an unrealistic target to achieve in absence of quality availability and accessibility of family planning services for male and pragmatic development for vasectomy in the country. In this context, the present work attempts to understand the situation of male sterilization and available policy and programmes encouraging vasectomy in India.

## Discussion and policy implications

**Fig. 1:**
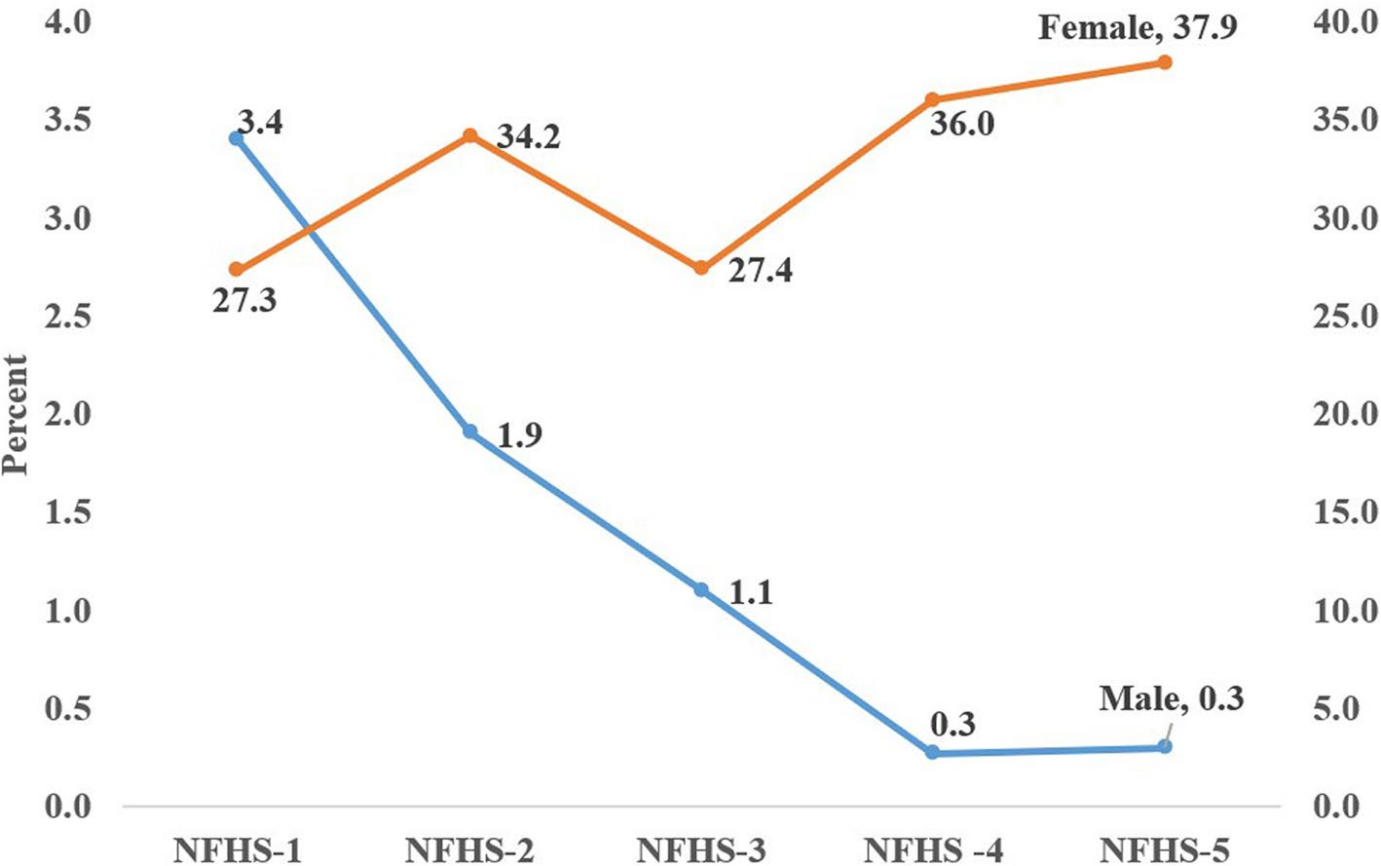
Trend of sterilization among male and female in India (NFHS)

In the world, nearly 1.1 billion women need family planning services out of 1.9 billion women who are in reproductive age (15–49 years) and 44 percent of them use modern contraceptive methods [11]. Estimations of United Nations highlighted that the only 2 percent male sterilizations performed against the 24 percent female sterilization worldwide. It also revealed that vasectomy prevalence remains highest in the United Kingdom of Great Britain (10.4%), followed by Republic of Korea (9.5%), Bhutan (8.0%), Australia (7.7%), Switzerland (4.9%), Nepal (4.6%), United States of America (4.3%), Canada (3.8%), Belgium (3.7%), Ireland (3.5%), Brazil (2.6%), Iran (2.1%) [11]. The current trends of vasectomy use call for an inspection of means and measures within the programme to achieve 30 percent vasectomy as mandated by the NHP-2017. The five rounds of NFHS highlighted that the use of male sterilization has been steadily decreasing in all states of India during the past three decades. For instance, the NFHS-1 reported that 3.4 percent of males had a vasectomy in India [7], which subsequently decreased over the four rounds from 1.9 percent in NFHS-2 [8] to 1.1 percent in NFHS-3 [12], 0.3 percent in NFHS-4 [13], and 0.3 in NFHS-5 [9] (Fig. 1). This decline has a varied manifestation in individual states like Andhra Pradesh (6.6%), Kerala (6.5%), and Maharashtra (6.2%), having the highest prevalence of vasectomy compared to other states in NFHS-1. While in the NFHS-2 and 3, male sterilization was highest in Himachal Pradesh, Andhra Pradesh, and Sikkim, its prevalence substantially decreased in Maharashtra, Gujarat, Haryana and Madhya Pradesh in the subsequent NFHS rounds. Himachal Pradesh (3.3%) and Telangana (2.0%) are two states of India where the acceptance of vasectomy actually increased between NFHS-4 to NFHS-5 (Table 1). Other than this, the remaining states have less than 1 percent of male sterilization in NFHS-5. It has also been evidenced that individual-level communication by health workers and peer group discussion plays a significant role in persuading males to vasectomy. The male population is often considered to be the underserved population in India in the context of involvement in the reproductive and maternal health care of women. Their contribution is negligible in ensuring the reproductive health of their spouse and the majority of the men are unaware of their role and responsibilities is ensuring the safe passage of reproductive years of the life of their women. However, evidence suggests that the substantial male population in rural tribal areas opted for vasectomy compared to their female counterparts owing to a conditional cash incentive scheme [14].

**Table 1:** Male sterilization in India

States and Union Territories	NFHS-1 (1992–93)	NFHS-2 (1998–99)	NFHS-3 (2005–06)	NFHS-4 (2015–16)	NFHS-5 (2019–21)
Andhra Pradesh	6.6	4.3	2.9	0.6	0.4
Arunachal Pradesh	0.4	0.1	0.1	0.0	0.0
Assam	2.3	1.0	0.2	0.1	0.1
Bihar	1.3	1.0	0.6	0.0	0.1
Chhattisgarh^a^	-	-	3.3	0.7	0.8
Goa	1.0	0.4	0.1	0.0	0.0
Gujarat	3.5	2.3	0.6	0.1	0.2
Haryana	5.0	2.1	0.7	0.6	0.9
Himachal Pradesh	3.2	7.3	6.3	2.4	3.3
Jammu and Kashmir	4.4	2.7	2.6	0.4	0.3
Jharkhand^a^	-	-	0.4	0.2	0.3
Karnataka	1.5	0.7	0.2	0.1	0.0
Kerala	6.5	2.5	1.0	0.1	0.1
Madhya Pradesh	5.1	2.2	1.3	0.5	0.7
Maharashtra	6.2	3.7	2.1	0.4	0.4
Manipur	2.9	1.1	0.5	0.1	0.0
Meghalaya	0.6	0.0	0.1	0.0	0.0
Mizoram	0.1	0.1	0.0	0.0	0.0
Nagaland	0.1	0.0	0.0	0.0	0.0
Odisha	3.4	1.7	1.0	0.2	0.3
Punjab	2.5	1.6	1.2	0.6	0.5
Rajasthan	2.4	1.5	0.8	0.2	0.3
Sikkim	-	2.4	4.5	3.4	1.7
Tamil Nadu	2.0	0.8	0.4	0.0	0.1
Tripura	2.4	-	0.5	0.0	0.0
Uttar Pradesh	1.4	0.7	0.2	0.1	0.1
Uttarakhand^a^	-	-	1.8	0.7	0.7
West Bengal	4.3	1.8	0.7	0.1	0.1
Telangana^a^	-	-	-	1.6	2.0
**Union Territories**					
Andaman and Nicobar Island^b^	-	-	-	0.0	0.2
Chandigarh^b^	-	-	-	1.3	0.3
Dadra and Nagar Haveli^b^	-	-	-	0.0	0.2
Daman and Diu^b^	-	-	-	0.0	-
Lakshadweep^b^	-	-	-	0.0	0.0
Delhi^b^	3.2	2.3	0.8	0.2	0.2
Puducherry^b^	-	-	-	0.0	0.3
Ladakh^a^	-	-	-	-	0.4
**INDIA**	**3.4**	**1.9**	**1.1**	**0.3**	**0.3**

Authors have compiled results of five consecutive National Family Health Survey from 1992 to 2021, ^a^ States were formulated in 2000, except Telangana, which was curved in 2014, and Ladakh was formulated in 2019. ^b^ Union Territories were excluded from the NFHS 1 to NFHS 3

To encourage people to adopt a permanent method of family planning, the government of India has implemented the conditional cash incentive scheme since 1981. This amount was supposed to compensate for peoples’ loss of wages during the treatment days at the health facility. The Government has given the flexibility to states/UTs to decide the amount to be paid to beneficiaries in their respective states. Incentives vary by state; a person who adopts vasectomy is eligible to receive ₹1500 under the “Family Planning Indemnity Scheme” – 2007 on the condition that the surgery should be conducted in a public health facility or approved private health facility. This amount used to be distributed among beneficiaries and other medical staff, including vasectomy acceptor (₹1100), surgeon (₹100 per surgery), staff nurse (₹15 per case), a motivator (₹200 per case), OT technician/helper (₹15 per surgery), refreshment (₹10 per surgery), camp management (₹10 per surgery) irrespective of high focus or non-high focus states. Apart from this, some states/UTs have an additional arrangement of payment in case of death (₹50,000), incapacitation (₹30,000), and for the treatment of serious post-operation complications (₹20,000) [15]. The government is actually providing more cash incentives for the vasectomy as against the tubectomy. For instance, incentives for the vasectomy operation is ₹1500 compared to the female sterilization operation which is ₹1000 [4]. It has been observed that the frontline workers play key role in persuading females to the health facilities for sterilization and not the amount of incentive offered. Although the government has incentivised the vasectomy method, people are reluctant to adopt it because of its coercive history, social stigma, ignorance, myths and misconceptions about the method, etc.

The current profile of contraceptive share between males and females conveys a clear male aversion to shoulder contraceptive responsibility. The NFHS-3 findings based on perceptions of males about family planning revealed that one-fifth (21.8%) of men aged group 15–54 positively affirmed the statement that contraceptive use is the women’s business and men should not worry about it. The perception of men regarding contraceptive use as a shared responsibility needs to be addressed through all the possible means of communication. Specific Information, Education, and Communication (IEC) materials need to be developed as regards the promotion of vasectomy, pronouncing its safety and benefits like medically safer, easier, less expensive, and most effective with the person’s discharge on the same day following the intervention [16]. The result of study indicates that the attitude towards utilization of vasectomy is largely shaped by the building proper knowledge on vasectomy and quality services available through program campaigns. And therefore, the visibility and prominence of vasectomy in IEC content would go a long way in defining the use of family planning services for male. In this context, the Indian Urologist justified that the “Vasectomy is as much an IEC operation as a surgical operation” [17]. On the other hand, female sterilization is a more complicated, time-consuming, and expensive procedure. The government can campaign for technological advancement in surgical processes which make vasectomy painless and bloodless surgery nowadays (Non-Scalpel Vasectomy (NSV)). The NSV is a simple operation that involves tying, cutting, and removing a portion of vas-deferential tubes which carry sperm from the testes.

Vasectomy has been the well-known contraceptive method of family planning since the 1956 in India. In fact, during the early days, about 77 to 80 percent of permanent sterilizations were male sterilization in India. This massive vasectomy camp approach was one of the main reasons for higher male sterilization during the political emergency imposed by the government during the years 1975–77 in India (Supplementary table 1). However, presently in India, the most commonly practised contraceptive methods have become gender biased and more female-centric, including permanent sterilization, Copper-T, intra-uterine contraceptive device (IUCD), combined oral pill cycles, emergency contraceptive pills, female condoms, and recently introduced injectable contraceptive, etc. (Table 2). There was considerable resistance to include injectable contraceptives within the family planning services; however, the advocacy failed to make a substantial effect on the implementation of the programme [18]. In contrast, male methods are limited to two, i.e., condoms and vasectomy, besides traditional methods like withdrawal and abstinence. Such imbalance in the availability of contraceptive options and lack of shared responsibility of contraception between men and women makes fertility regulation women-centric. This need not be taken lightly, given its bearing on women’s bodies and the sacrifice made thereof to realise the greater social good of low fertility. The availability of an extensive medical army, including, male multipurpose workers (MPWs), Auxiliary Nurse Midwife (ANM), Accredited Social Health Activist (ASHA), and Male/ Female Mid-Level Health Persons (MLHP), can be used to play a curial role in providing a basket of family planning methods in the community. And they can be encouraged by providing additional incentives to make community participation more vibrant, specifically emphasising male involvement in utilizing the family planning methods. The unavailability of quality vasectomy services at the health facilities must be considered as much of a gender issue besides being a programme concern [19]. There is consistent and sufficient evidence to indicate that women are disproportionately burdened with contraceptive utilization, and more or less, the family planning services are largely women-centric in India. This clearly is an outcome of the absence of policies and programmes to target men towards promoting vasectomy, which is revealed in a consistent reduction in the share of vasectomy prevalence. Available studies highlight that the male-centric focus resulted in greater vasectomy use in Nepal (7·8% prevalence), Brazil (5·1%), and Colombia (3·4%) [20]. The developing countries mainly treated unavailability of vasectomy services is not only the gender issue but at the same time programme issue and addressing it resulted in substantial higher prevalence of vasectomy in Canada (22%), North America (12%), United Kingdom (17%) and New Zealand (21%). Australia, Belgium, Denmark, Spain and USA [19]. Henceforth, the absence of a genuine promotion of vasectomy, the set goal under the NHP of increasing vasectomy prevalence in a time-bound manner may well remain beyond reach. This would not only ease women of the contraceptive burden but also reduce contraceptive-linked morbidity in women.

**Table 2:** Contraceptive use reported by women aged 15‒49 in India (*in percentages*)

Contraceptives methods in India	NFHS–I (1992–93)	NFHS–II (1998–99)	NFHS–III (2005–06)	NFHS–IV (2015–16)	NFHS–V (2019–21)
Female Sterilization	27.4	34.2	37.3	36	37.9
Male Sterilization	3.5	1.9	1.0	0.3	0.3
Pill	1.2	2.1	3.1	4.1	5.1
Intrauterine Device (IUD)	1.9	1.6	1.7	1.5	2.1
Injectable Contraceptives	0	-	0.1	-	0.6
Condoms	2.4	3.1	5.2	5.6	9.5
Any modern Method^a^	36.5	42.8	48.5	47.8	56.5
Any methods^b^ (Including traditional)	40.7	48.2	56.3	53.5	66.7

*NFHS* National Family Health Survey

^a^Any modern method includes other modern methods that are not shown separately; ^b^Any methods include other methods that are not shown separately

## Supplementary Information

Additional file 1: Supplementary Table 1. Sterilization in India since 1966.

## Data Availability

The data used in this study are publicly available at https://dhsprogram.com/data/

## Footnotes

1 Cafeteria approach is “basket of choices’’ of family planning methods available in the public health facilities including female sterilisation, male sterilisation, intrauterine contraceptive device (IUCD), oral contraceptives, injectable and condoms.

## References

[1] Government of India. National health policy. New Delhi: Ministry of health and family welfare; 2017.

[2] Government of India. Annual Report. New Delhi: Ministry of Health & Family Welfare; 2020. https://mohfw.nic.in.

[3] Dyson T (2018). A Population History of India.

[4] Government of India. Reference manual for male sterilization. Family planning division. New Delhi: Ministry of health and family welfare government of India; 2013.

[5] Government of India, family welfare programmes in India, ministry of health and family welfare, year book:1989–1990. New Delhi: Department of family welfare; p. 1–362.

[6] Bhende AA, Kanitkar T (1978). Principles of Population Studies.

[7] International Institute for Population Sciences (IIPS) (1995). National Family Health Survey-1, India 1992–93.

[8] International Institute for Population Sciences (IIPS) (2000). National Family Health Survey-2, India, 1998–99.

[9] International Institute for Population Sciences (IIPS) (2021). National Family Health Survey-5, India 2019–21.

[10] HMIS. Health management information system. Ministry of health & family welfare, government of India; 2021. https://hmis.mohfw.gov.in/#!/.

[11] United nations, department of economic and social affairs, population division (2019). Contraceptive use by method 2019: Data Booklet (ST/ESA/SER.A/435).

[12] International Institute for Population Sciences (IIPS) (2007). National Family Health Survey-3, India 2005–06.

[13] International Institute for Population Sciences (IIPS) (2017). National Family Health Survey-4, India 2015–16.

[14] Jungari S, Paswan B (2019). Male Perception and Participation in Family Planning Among Tribal Communities of Maharashtra, India: A Mixed-Method Study. Int Q Community Health Educ.

[15] Government of India. Manual for family planning indemnity scheme. Family planning division. New Delhi: Ministry of health and family welfare, government of India; 2016.

[16] Farley TM, Meirik O, Mehta S, Waites GM (1993). The safety of vasectomy: recent concerns. Bull World Health Organ.

[17] Subramanian L, Cisek C, Kalanisis N, Pile JM: The Ghana vasectomy initiative facilitating client –provider communication on noscalpel vasectomy, Patient Educ Couns. 81(3)374–80, dol: 10.1016/j.pec. 2010.05.008. Epub 2010 10.1016/j.pec.2010.05.008 21129618

[18] Nair S (2017). Injectable Contraceptives. Econ Polit Wkly.

[19] Jacobstein Roy. The kindest cut: global need to increase vasectomy availability; 2015;3(12):e733–34. 10.1016/S2214-109X(15)00168-0. 10.1016/S2214-109X(15)00168-0 26545447

[20] Roy Jacobstein and John Pile. Vasectomy: the unfinished agenda: the ACQUIRE project working paper, 2007. New York, NY 10001 U.S.A. https://www.acquireproject.org.

