# Supplementary material for: Disappearing male sterilization in India: do we care?
Source: Contracept Reprod Med. 2023 May 4;8:31. doi: 10.1186/s40834-023-00228-w (PMC10157918; doi:10.1186/s40834-023-00228-w)
Supplement: Supplementary file 1 — Additional file 1: Supplementary Table 1. Sterilization in India since 1966. [file 40834_2023_228_MOESM1_ESM.docx]

| **Supplementary Table 1: Sterilization in India since 1966** | | | | |  |
| --- | --- | --- | --- | --- | --- |
| **Year** | **Vasectomy (percent)** | **Vasectomy** | **Tubectomy** | **Total sterilization** |  |
| **1966-67** | **88.5** | 7,85,378 | 1,01,990 | 8,87,368 |  |
| **1967-68** | **89.6** | 16,48,152 | 1,91,659 | 18,39,811 |  |
| **1968-69** | **83.1** | 13,83,053 | 2,81,764 | 16,64,817 |  |
| **1969-70** | **74.2** | 10,55,860 | 3,66,258 | 14,22,118 |  |
| **1970-71** | **66.1** | 8,78,800 | 4,51,114 | 13,29,914 |  |
| **1971-72** | **74.1** | 16,20,076 | 5,67,260 | 21,87,336 |  |
| **1972-73** | **83.7** | 26,13,263 | 5,08,593 | 31,21,856 |  |
| **1973-74** | **42.8** | 4,03,107 | 5,39,295 | 9,42,402 |  |
| **1974-75** | **45.2** | 6,11,960 | 7,41,899 | 13,53,859 |  |
| **1975-76** | **53.9** | **14,38,337** | **12,30,417** | **26,68,754** | **Government of India has imposed political emergency from 1975 to 1977** |
| **1976-77** | **75.0** | **61,99,158** | **20,62,015** | **82,61,173** |  |
| **1977-78** | **19.8** | 1,87,609 | 7,61,160 | 9,48,769 |  |
| **1978-79** | **26.3** | 3,90,922 | 10,92,985 | 14,83,907 |  |
| **1979-80** | **26.6** | 4,72,687 | 13,05,237 | 17,77,924 |  |
| *Source: Government of India, Family Welfare Programme in India, Ministry of Health and Family Welfare, Year Book:1989-1990, Department of Family Welfare, New Delhi, Page1-362* | | | | |  |
